# Best Practices for Ethical Sharing of Individual-Level Health Research Data From Low- and Middle-Income Settings

**DOI:** 10.1177/1556264615594606

**Published:** 2015-07

**Authors:** Susan Bull, Phaik Yeong Cheah, Spencer Denny, Irene Jao, Vicki Marsh, Laura Merson, Neena Shah More, Le Nguyen Thanh Nhan, David Osrin, Decha Tangseefa, Douglas Wassenaar, Michael Parker

**Affiliations:** 1 University of Oxford, UK; 2 Mahidol University, Bangkok, Thailand; 3 University of KwaZulu-Natal, Pietermaritzburg, South Africa; 4 KEMRI Wellcome Trust Research Programme, Nairobi, Kenya; 5 Oxford University Clinical Research Unit, Ho Chi Minh City, Viet Nam; 6 Society for Nutrition, Education and Health Action, Mumbai, India; 7 Children’s Hospital 1, Ho Chi Minh City, Viet Nam; 8 University College London, UK; 9 Thammasat University, Bangkok, Thailand

**Keywords:** biomedical research ethics, data sharing, data release, research data, research governance, low-income countries, middle-income countries, clinical research, health policy, privacy, trust

## Abstract

Sharing individual-level data from clinical and public health research is increasingly being seen as a core requirement for effective and efficient biomedical research. This article discusses the results of a systematic review and multisite qualitative study of key stakeholders’ perspectives on best practices in ethical data sharing in low- and middle-income settings. Our research suggests that for data sharing to be effective and sustainable, multiple social and ethical requirements need to be met. An effective model of data sharing will be one in which considered judgments will need to be made about how best to achieve scientific progress, minimize risks of harm, promote fairness and reciprocity, and build and sustain trust.

Data-sharing is increasingly seen as an important requirement for effective and efficient biomedical research by researchers and research funders. It is noteworthy, however, that many of the arguments presented for greater sharing of data are grounded in empirical claims about its potential to generate more and better science and to enable more efficient use of research resources for the greater good. In principle, these claims are amenable to testing and evaluation. It may be that some approaches to data sharing are more likely to promote effective and efficient research than others, and it is also possible that the most effective and sustainable approach may vary between settings and different forms of data or research. One factor in the sustainability of any approach to data sharing is likely to be the extent to which it is compatible with enduring collaborative partnerships between researchers in their capacity as data producers and/or data users. Another related factor is likely to be the extent to which proposed approaches to data sharing reflect a reasonable alignment between the *mandate* for data-sharing claimed, often in the “public interest” by researchers, research funders and journals, and the *social license* important stakeholders, including research participants, communities, research ethics committees, and the wider public would consider themselves to have given for data sharing (Carter, Laurie, & Dixon-Woods, 2015).

Where the social license for sharing individual-level data from clinical and public health research and the mandate claimed by research actors are reasonably well-aligned, there may be grounds for confidence that more effective data sharing will indeed lead to more successful and appropriate scientific research. Where, however, there is a significant gap between them, it seems less likely that the widespread adoption of data-sharing practices will be supported without significant investment of time and resources into the building of trust and confidence. Moreover, where there is a lack of support from the public, key data governors (such as research ethics committees), researchers, and research institutions, there is good reason to hypothesize that research progress may be undermined by lack of trust and/or confidence.

While important in all research settings, such considerations are likely to be particularly important, both morally and instrumentally, in research conducted in low- and middle-income settings where social justice concerns are at their most acute. Against this backdrop, significant progress in data sharing, even where it is believed to be capable of contributing to scientific knowledge addressing the global burden of disease, is likely to require the development of models of good data-sharing practice capable of commanding the trust and confidence of relevant stakeholders. These will need to be grounded in shared understandings of what is required for data sharing to be “equitable, ethical, and efficient” (Walport & Brest, 2011). Careful consideration of stakeholders’ differing interests in the development of governance policies and processes is needed to enable sound judgment of the balance between the need to share data in a way that maximizes their use, while ensuring that appropriate protections are in place to minimize potential harms (Parker et al., 2009).

In this article, we report on the results of a literature review and qualitative studies conducted in five low- and middle-income settings, which explored the experiences and views of key stakeholders about best practices in sharing individual-level data from clinical and public health research (Bull, Roberts, & Parker, 2015; Cheah et al., 2015; Denny, Silaigwana, Wassenaar, Bull, & Parker, 2015; Hate et al., 2015; Jao et al., 2015; Merson et al., 2015; Parker & Bull, 2015). It is noteworthy that the systematic review was unable to identify any empirical studies of stakeholders’ views about best practices in sharing individual-level data from clinical and public health research in low- and middle-income settings. Before discussing the findings of our own research, we summarize the main arguments in favor of, and concerns about, data sharing in the literature.

## The Potential Benefits of Data Sharing

It is increasingly widely believed that the greater sharing of de-identified individual-level public health and medical research data would be of high value. Such sharing has the potential to enable verification, replication, and expansion of research results and to provide means of addressing biases, deficiencies, and dishonesty in published and unpublished research (Chan, Hróbjartsson, Haahr, Gøtzsche, & Altman, 2004; Chan et al., 2014; Rodwin & Abramson, 2012; Ross, Lehman, & Gross, 2012; Smith, 1994; Wieseler, McGauran, Kerekes, & Kaiser, 2012). It is also claimed that shared data sets have the potential to generate and enable the addressing of novel research questions, inform the design of future research, contribute to powerful meta-analyses, and to support the building of capacity in analysis (Anderson & Merry, 2009; Dove, Knoppers, & Zawati, 2014; Manju & Buckley, 2012; Vallance & Chalmers, 2013). It is argued that researchers and research institutions may benefit from sharing data, as the visibility and relevance of their research increases, potentially leading to increased collaborations and research funding (Pisani, Whitworth, Zaba, & Abou-Zahr, 2010a; Piwowar et al., 2008).

These arguments are important because, if the empirical claims upon which they are based are true, they suggest that sharing data has the potential to make an important contribution to scientific progress and improved health. This would be achieved by expanding the knowledge base used to inform not only research but also the ethical review of research, health care policy development, purchasing decisions, regulatory review of novel treatments, and clinical care (Gotzsche, 2011; Haines & Gabor Miklos, 2011; Wieseler et al., 2012).

In addition to these important claims, which have an ethical dimension, several additional explicitly moral arguments have been made for greater data sharing. These include arguments about the ethical importance of maximizing the value and utility of data sets and of minimizing the costs and burdens of unnecessary duplication of research (Doshi, Goodman, & Ioannidis, 2013; Rani, Bekedam, & Buckley, 2011). Data sharing is considered to enable the best use of valuable resources, and to appropriately honor the contributions of research participants (Gotzsche, 2012; Langat et al., 2011; Mello et al., 2013; Sommer, 2010; Strech & Littmann, 2012). Moreover, it is argued that the interests of the wider public, who when asked tend to support research, also warrant treating data as a public good to be shared (Tangcharoensathien, Boonperm, & Jongudomsuk, 2010; Toronto International Data Release Workshop Authors, 2009).

## Concerns About Data Sharing

While the potential value of data sharing is increasingly recognized, several important enduring concerns need to be addressed for data sharing to be both successful and appropriate. The literature suggests that many of these concerns are scientific. As with primary research, the results of secondary research may be flawed if analysis is inappropriate or biased, or if reanalyses are based on misinterpretations of the data or are conducted to push specific agendas (Pearce & Smith, 2011; Wieseler et al., 2012). The concern is that this may be more likely in secondary research where those who use the data are removed from the context of data collection and curation. Concerns also arise from the belief that effective data sharing is resource intensive. Curating data for sharing and developing appropriate data-sharing policies and processes takes significant expertise, effort, and resources (Mello et al., 2013; Nisen & Rockhold, 2013; Whitworth, 2010). Monitoring uses of secondary data, responding to queries from data accessors, and evaluating results of secondary research will also require appropriate resourcing.

Other concerns relate to the impact of data sharing on the researchers who collect data and produce shared data sets. Mandated data sharing could affect researchers’ abilities to publish primary findings from their studies, and to file relevant patents, which might in turn create disincentives to fund and conduct primary research (Mello et al., 2013; Savage & Vickers, 2009; Vickers, 2006). A lack of clarity about ownership rights and intellectual property issues may make it difficult to determine who has the authority to decide how data should be shared (Anderson & Merry, 2009; Manju & Buckley, 2012; Mello et al., 2013). Primary researchers have also expressed concerns about reputational damage if their analyses and findings are challenged in secondary research (Savage & Vickers, 2009; Smith, 1994). Perhaps the most commonly expressed concern, however, is that primary researchers’ scientific activities may be undermined by data sharing in situations where secondary users, who may be better resourced, are able to utilize data more quickly than those who produced it. Given the importance of peer-reviewed publications in funding decisions and academic advancement, sufficient opportunities for primary researchers to publish the findings of research are critical (Coady & Wagner, 2013; Rathi et al., 2012; Ross et al., 2012), as is appropriate recognition for producing and curating data sets for sharing (Langat et al., 2011; Pisani & AbouZahr, 2010).

Finally, and importantly, concerns have been expressed about the potential for data sharing to harm or to fail to respect the interests of participants. A major concern is to ensure that research participants’ privacy is protected and the confidentiality of identifying data is preserved while maintaining the utility of data sets (Castellani, 2013; Sieber, 2006; White, 2013). Policies and processes for de-identifying data continue to develop (de Wolf, Sieber, Steel, & Zarate, 2006; Hughes, Wells, McSorley, & Freeman, 2014; Zarin, 2013), but the possibility of identifying participants, directly or deductively, particularly when data sets may be combined, remains of concern (European Medicines Agency, 2014; Goldacre, 2013). In addition to these concerns, there has also been much discussion about the implications of data sharing for consent. There is some debate about the need to inform participants that de-identified data will be shared (de Wolf, Sieber, Steel, & Zarate, 2005; Gotzsche, 2011; Greenhalgh, 2009), and, if so, how best to notify participants about the possible ramifications of sharing (Mello et al., 2013; Nisen & Rockhold, 2013; Toronto International Data Release Workshop Authors, 2009). Concerns have been raised that if data are released without explicit approval from participants, the delicate trust relationship between researchers and participants will be harmed (Greenhalgh, 2009; Pearce & Smith, 2011; Piwowar et al., 2008). There is broad agreement that consent is required, but some disagreement about what this implies. Broad consent to data sharing is becoming increasingly common and accepted (Nisen & Rockhold, 2013; Pisani & AbouZahr, 2010), but some commentators have argued that explicit and dynamic consent for all research uses on each occasion is preferable (Kaye et al., 2015).

## Data Sharing in Low- and Middle-Income Settings

Most published literature on data sharing focuses on research in high-income settings. However, although the literature is limited, versions of the arguments above have also begun to appear in literature concerning research in low- and middle-income settings (Manju & Buckley, 2012; Sankoh & Ijsselmuiden, 2011; Tangcharoensathien et al., 2010). Some of this discussion has arisen in the context of the growth of genomic research in low-income settings (Parker et al., 2009) and increasingly, particularly in response to the recent Ebola outbreak in West Africa, calls have been made for facilitating timely data sharing to inform responses to public health emergencies and disease outbreaks (Langat et al., 2011; Yozwiak, Schaffner, & Sabeti, 2015).

While many of the above arguments are relevant to the sharing of research data in any setting, there are some significant differences. For example, while limited resources may be a hindrance to data sharing in higher income settings, they are likely to be a significant barrier to ethical data sharing in lower income settings (Manju & Buckley, 2012; Rani et al., 2011; Sankoh & Ijsselmuiden, 2011; Tangcharoensathien et al., 2010; Walport & Brest, 2011; Whitworth, 2010). For high-quality individual-level data to be shared in databases with long-term sustainability, significant investment in human resources, technology, and infrastructure will be required. Training, mentoring, and career pathways also need to be provided for a range of specialist support staff to document and curate data sets and manage data-release processes.

Furthermore, the arguments about the potential impact of data sharing on primary data producers are also likely to be much more pressing in the case of researchers and research institutes in low- and middle-income settings. In such settings, differences in capacity between primary data producers and secondary data users, most likely in high-income settings, who may conduct very rapid analyses of the data, are much greater.

It is against this background that we conducted a multisite empirical ethics research project using qualitative research methods to examine stakeholder experiences of, and views about, best practices in sharing individual-level data from clinical and public health research. Interviews and focus groups were conducted with key stakeholders, including health professionals, researchers, and community representatives in India, Vietnam, Thailand, South Africa, and Kenya. The findings of the individual studies are published as separate articles in this special issue (Cheah et al., 2015; Denny, Silaigwana, Wassenaar, Bull, & Parker, 2015; Hate et al., 2015; Jao et al., 2015; Merson et al., 2015). In what follows in this article, we present an overarching analysis of the findings of the project as a whole. While this analysis is not easily generalizable to other low- and middle-income settings, we believe it offers some interesting and important insights which are likely to be of use to researchers, research ethics committees, funders, and policy makers. It also suggests a number of potential avenues for research in these and other settings. In the sections below, we present our findings under two broad headings. The first is an overview of four key themes arising from our analysis relating to views about important requirements for good ethical data-sharing practice. The second identifies four ways forward in thinking through the practicalities of good data-sharing practice.

## Core Considerations in Ethical Data Sharing

Taken together, the analyses of the five individual studies suggest four key factors as important considerations in judging whether any particular data-sharing initiative is likely to be an example of good data-sharing practice and likely to command support in the development of models of data-sharing practice. These are the value of data sharing, minimizing harm, promoting fairness and reciprocity, and trust.

**Box 1:** 

Key considerations in good data-sharing practice:
The value of data sharing
Minimizing harm
Promoting fairness and reciprocity
Trust

### The Value of Data Sharing

Echoing a broad consensus in the published literature, there was general support at all five empirical study sites for data sharing among the stakeholders, particularly among senior and junior researchers. Attitudes of community members and participants were typically more cautious, although support for data sharing often grew as they became familiar with the concepts involved, the potential advantages of sharing, and safeguards that could be implemented to address concerns. What this suggests is that for all stakeholders, an assessment of the potential benefits of data sharing is likely to be an important factor in the question of whether or not it constitutes an example of good data-sharing practice. It is important to note, however, that this is not the overriding consideration. The benefits of data sharing are seen as one factor in any such judgment. In the following, we discuss stakeholders’ views about the other factors seen as important requirements for best practice in data sharing.

### Minimizing Harm

Concerns about minimizing harms of research focused on ensuring that participants’ interests were not adversely affected when individual-level data were shared. At all sites, protecting participants’ privacy and ensuring that identifying data remained confidential were considered to be of key importance by all stakeholders, reflecting a consensus in the reviewed literature (Bull, Roberts & Parker, 2015). However, reflecting the broader discussions of harm in the literature outlined above, de-identification of data was not necessarily considered sufficient in itself to minimize the risk of harm. Risks of harm were associated both with the sensitivity of the data sets collected and with the uses that could be made of the data. Participants described a wide range of data sets that were likely to be sensitive, but noted that all data could potentially be sensitive, which made it important to understand both the context in which data had been collected, and proposed secondary uses of it.

Particular concerns were raised about secondary research with de-identified data contributing to the stigmatization of identifiable communities, populations, and even countries. Data and secondary research about topics such as disease prevalence and socioeconomic status had the potential to increase stigmatization when results were insensitively reported, or when secondary users had vested interests. In some cases, more direct harms could potentially result from data sharing, for example, if data about participants in impoverished informal settlements collected for public health research purposes was accessed by secondary users to inform accelerated resettlement.

In addition to potential harms to participants, researchers at all sites echoed concerns in the literature discussed above about the potential for data sharing to adversely affect research capacity and career development in low- and middle-income settings. Concerns were raised, for example, about primary researchers being side-lined by better resourced institutions conducting secondary research. Secondary users’ critiques of the quality of data sets and of the primary research could affect primary researchers’ reputations, ability to attract research funding, and career development. If insufficient resources were available for appropriate preparation and curation of data sets prior to mandatory sharing, and to respond to secondary users’ queries during such sharing, then resources might be wasted in secondary analyses which were unable to generate valid answers to the research question.

### Promoting Fairness and Reciprocity

At all sites, the importance of ensuring that data-sharing practices did not increase existing inequalities was considered fundamental, reflecting discussions in the literature about the need for data sharing to be ethical and equitable. Participants focused not only on the importance of protecting stakeholders from harm but also promoting their relevant interests. In such discussions, community stakeholders noted that their contributions to the development of a valuable resource suggested that the resource should be used to directly or indirectly benefit their community. There was discussion about the possibility of direct benefits accruing to participants from secondary research, but no general consensus that direct benefits were a requirement of ethical data sharing. Community stakeholders in multiple settings discussed the importance of secondary research providing indirect benefits, such as addressing health issues of relevance to their communities. If such issues were not to be addressed, then it was considered important that secondary research should have the potential to advance health more generally.

Researchers’ perspectives were similar to those highlighted in the literature, emphasizing the importance of ensuring that data sharing was conducted in a way that did not adversely affect their careers or ability to conduct health research of relevance to the communities in which they were based. Research data were perceived to be a valuable resource created and managed with considerable effort. The importance of these data was not limited to their value in addressing specific research questions of relevance to communities, but also linked to their potential to leverage funding and capacity development for future research. Echoing discussions in the literature, a core requirement of fair data sharing was that primary data producers received appropriate recognition of their role in producing valuable data sets. Capacity building may be needed to support researchers’ effective and timely analyses of their own data sets, to enable effective participation in larger collaborations analyzing multiple linked data sets, and to enable identification and secondary analysis of relevant data sets to address topics of interest. Some stakeholders went further and suggested that principles of reciprocity require that data producers should be routinely offered the opportunity to be involved in secondary analyses of their data, with appropriate acknowledgment of this additional contribution.

### Trust

The final core theme that arose in conversations with stakeholder groups at all sites was the importance to participants, communities, researchers, and the wider public of ensuring that data were shared in both a trusted and trustworthy manner, reflecting discussions about sample and data sharing in the literature (Erlich et al., 2014; Kaye, Heeney, Hawkins, de Vries, & Boddington, 2009; Murtagh et al., 2012; Tindana, Molyneux, Bull, & Parker, 2014). Data sets, primary researchers, secondary data users, and data-sharing policies and processes all needed to be trusted for effective and ethical data sharing. Stakeholders noted that governance policies and processes for data sharing could promote trust by ensuring that proposals for secondary access were evaluated by parties trusted to identify potential harms to stakeholders and to promote their interests. Mechanisms for building trust when developing and implementing data-sharing policies and practices included engagement with government health authorities, community consultation, stakeholder representation, and providing feedback about the results of data sharing.

Researchers highlighted the need for data-sharing policies and processes that would permit them to fulfill their obligations to research participants. In addition, trust needed to be built that the production and sharing of data sets would be appropriately recognized and rewarded in future career development and funding applications. Stakeholders noted that secondary researchers would need to be trusted to comply with the conditions under which they were granted access to the data sets and that enforcement of data access conditions could be challenging.

Stakeholders’ concerns about trustworthiness were often proportionate to their levels of concern about harms and fairness in data sharing. For data sharing to be ethical, it needed to be shared in ways that minimized harm and appropriately promoted participants’ interests. At one extreme, stakeholders noted that when research was conducted on sensitive topics, research participants were only likely to provide accurate data to primary researchers whom they trusted. They might hold an expectation that the data would remain confidential to the research team. In such cases, careful consideration of whether data sets could be shared at all would need to be undertaken, and, if so, of what governance measures would promote and sustain trust. At the other extreme were data sets considered to be less sensitive, such as subsets of clinical measurements relied on in published research articles. There could be less need to scrutinize and control uses made of such data sets, and they might even be released via open access mechanisms.

## Ethical Data Sharing: Ways Forward

Having identified four factors seen by stakeholders as key considerations in the development of and judgment about good ethical practice in data sharing in low- and middle-income settings, we explore in more detail below some of the areas identified as building blocks of good practice: consent, governance processes, data-sharing policies, and approaches to capacity building.

### Seeking Consent to Data Sharing

There was substantial variation in views within and between sites about best practices in seeking consent to prospective data sharing. Two related topics emerged as core considerations: the ethical acceptability of broad consent to data sharing, and the nature and extent of information to be provided to participants about data sharing if consent is to be considered appropriately informed.

When seeking participants’ consent to sharing data in genetic and genomic research, approaches range from seeking broad consent at the inception of research (Mascalzoni, Hicks, Pramstaller, & Wjst, 2008) to using online platforms to enable participants to manage dynamic ongoing consent to sharing (Kaye et al., 2015; Mathews & Jamal, 2014). While views differ about whether requesting broad consent to unspecified future research is respectful of participants and can be sufficiently informed to be valid, it is clear that broad consent approaches are increasingly widely accepted (Caulfield, 2007; Sheehan, 2011). In this study, the ethical acceptability of broad consent to future research purposes attracted varying views from stakeholders within and between sites. Many arguments in favor of broad consent were pragmatic, with researchers referring to the difficulty and expense of recontacting research participants, perhaps repeatedly, for consent to specific secondary research proposals. Requirements to recontact participants for specific consent were also considered to have ethical implications in settings where web-based interfaces for ongoing management of consent to sharing were not practicable. In contexts where contacting participants to reconsent would need to be via shared telephones, or by physical visits from field staff, seeking specific consent could risk harming local participants by identifying them as having taken part in a specific study. Moreover, participants might not wish to be involved in ongoing decision making about secondary research and could find being contacted to reconsent burdensome and inconvenient.

There was some consensus that seeking broad consent to data sharing could be ethically acceptable, if accompanied by appropriate information in an accessible form at the time of consent and by effective and trusted data-sharing governance procedures, a similar finding to a review of biobank participants’ attitudes to consent (Simon et al., 2011). In some cases, broad consent to a range of research areas might be sought, such as research related to health care or to improving statistical methods, an approach taken in genomic research from low- and middle-income settings (Parker et al., 2009). While generally supportive of broad consent models, stakeholders noted that policies would need to be in place to determine what should happen if access to data was requested for different purposes from those discussed in the consent process, or if access was sought from specific groups, such as commercial organizations.

There was significant variation in views about the amount and type of information that should be provided to participants when seeking consent to data sharing. There was general consensus that, given participants’ differing levels of willingness to share data, if participants are to be respected, they must be informed that their data could be shared and given the opportunity to opt out of sharing if desired. Moreover, if information about sharing was withheld during consent and community engagement processes, and subsequently became known to research participants and communities, this could adversely affect trust in researchers and impact on willingness to take part in future research. Opinions about how much information should be provided ranged from explaining that data would be shared, but providing minimal further information, to providing explicit details of data-sharing plans and secondary analyses. Where it was considered important to provide more than minimal information, stakeholders discussed the importance of informing participants about why data sharing was proposed, what kinds of secondary users could request access, for what purposes, and the processes that would be used to govern data sharing.

Given the novelty of data sharing, and the range of opinions about the nature and extent of information that should be provided about sharing, further research is needed to determine what information should be provided to research participants in varying contexts when data sharing is anticipated. Insights gained during the development of data collection materials for this study, and from the views of stakeholders, point to the need for further research to determine how best to explain this complex and unfamiliar concept to participants in a comprehensible manner in conjunction with other information they receive about a proposed study.

### Governing Data Sharing

When considering how best to manage data sharing, researchers at each site expressed a strong preference for sharing data within collaborative relationships. This was the most familiar form of sharing for most researchers and considered important to enable them to fulfill obligations to minimize potential risks of sharing and appropriately promote participants’ interests. Researchers noted that sharing data within collaborations supported trust building and capacity development. In addition, collaboration could improve the quality of research by ensuring that the research context was understood and errors addressed, and could ensure appropriate recognition of researchers’ contributions to secondary research. The advantages of sharing clinical and public health data through collaborative data-sharing arrangements in a range of low- and middle-income settings have been recognized in the literature (Manju & Buckley, 2012; Tangcharoensathien et al., 2010; Whitworth, 2010). Given strong support for, and perceived advantages of, collaborative data sharing, we suggest that this issue be given careful consideration when developing policies and processes for data sharing in low- and middle-income settings. Further research into the benefits and disadvantages of such approaches would be valuable. Our findings suggest that the development of larger collaborative approaches to research as a context for data sharing might offer an interesting and novel mechanism for effective, trusted data sharing in low- and middle-income settings.

If data are to be shared with secondary researchers with whom there is no collaborative relationship, the views of stakeholders in this study were similar to those reported in the literature and emphasized the importance of transparent, accountable, efficient, fair, and proportionate governance processes (Bull, Roberts & Parker, 2015). Such processes were considered necessary to minimize potential harms of research and enable researchers to fulfill their obligations to research participants. Views differed between sites about the appropriate composition of governance groups. In some settings, it was considered appropriate for scientific and research ethics committees to review data-sharing proposals, while in others, the value of establishing novel data-sharing committees or appointing data managers was considered. Views also differed about which stakeholder interests should be represented in governance processes and how best to do so. Given the novelty of sharing data from clinical and public health research in the sites in this study, some stakeholders discussed the value of reviewing the differing approaches to governing data sharing currently in place in a range of settings, as well as guidelines for the constitution of governance procedures. Among all stakeholders, the importance of developing policies and processes to guide reasoned data-sharing decisions was emphasized, as discussed below.

### Data-Sharing Policies

To inform best practices in sharing data from clinical and public health research, stakeholders identified a number of areas where policies and standard operating procedures would be valuable. Priority topics to address included the following:

- Quality control and preparation of qualitative and quantitative data sets for sharing, including guidelines for de-identification of data.
- Preparation of metadata to accompany data sets, including metadata about the context in which the data were collected.
- The design and conduct of consent processes, including core information to be provided to research participants.
- Information that secondary researchers seeking to access data sets should provide.
- Conditions that secondary researchers should be obliged to comply with when accessing, analyzing, and reporting data, including acknowledgment of the primary data source and researchers.
- The composition and conduct of bodies overseeing data release.
- Guidelines for prioritization of data sets for release and criteria for determining how specific data sets should be released.
- Guidelines for case-by-case review of data-access applications where appropriate.

Stakeholders’ views about the importance of developing consensus standards for data sharing have been echoed in the literature (Manju & Buckley, 2012; Mello et al., 2013; Piwowar et al., 2008; Rani et al., 2011; Vallance & Chalmers, 2013). The value of developing national guidance for data sharing, to inform the development of institutional policies and processes, and of receiving clear guidance from research funders about data-release requirements and mechanisms has been recognized in the literature and by stakeholders in this study (Manju & Buckley, 2012; Toronto International Data Release Workshop Authors, 2009).

Current data-sharing policies and processes can potentially provide a valuable resource to inform the development of national guidance and institutional governance for data sharing in low- and middle-income settings (Alter & Vardigan, 2015; Herbst et al., 2015; Lötter & van Zyl, 2015). As outlined above, care will be needed to ensure that policies and processes can enable the identification of data sets and proposed secondary uses that are likely to be sensitive in specific research contexts, and ensure that harms are minimized and stakeholders’ interests appropriately promoted. In addition, funders and policy developers in higher income settings should be informed about the priorities and interests of stakeholders in low- and middle-income settings to ensure that the policies for application in such settings minimize harm, promote stakeholders’ interests, and build trust (Carr & Littler, 2015). There are some existing models in which the collaborative development of data-sharing policies and practice has been effective (Parker et al., 2009).

### Capacity Building

Researchers at all the sites emphasized the importance of building capacity and access to sufficient resources to support best practices in data sharing. Echoing published literature about capacity building for data sharing in low- and middle-income settings, multiple areas for capacity development and appropriate resourcing were identified (Manju & Buckley, 2012; Pisani & AbouZahr, 2010; Pisani et al., 2010a; Pisani, Whitworth, Zaba, & AbouZahr, 2010b; Rani et al., 2011; Sankoh & Ijsselmuiden, 2011; Tangcharoensathien et al., 2010; Walport & Brest, 2011; Whitworth, 2010). Stakeholders noted that if data sharing is to be ethical, resources and expertise must be available to enable appropriate implementation of all of the policy priorities listed above. In addition, attention must be paid to ensuring that the management of data sharing is not likely to undermine existing or emerging scientific and data analysis capacity, upon which, to a significant extent, the sustainable future of successful scientific research in such settings depends. Resources are also required for the development and maintenance of infrastructure required to support long-term storage and sharing of data, including responding to queries about data sets or supporting collaborative research. Expertise in curation and good data management will also need to be developed and supported. Principles of fairness and reciprocity also suggest that resources be made available to enable researchers in low- and middle-income settings to undertake secondary analysis of data sets that have the potential to address their research priorities.

## Concluding Thoughts

In this article, we have provided an overarching analysis of the findings of a multisite empirical research study into the views of key stakeholders in India, Kenya, Thailand, Vietnam, and South Africa about the key requirements for the development of sustainable models of good data-sharing practice in research in low-income settings. The findings of the individual studies are reported in detail elsewhere in this issue of *Journal of Empirical Research on Human Research Ethics* (Cheah et al., 2015; Denny, Silaigwana, Wassenaar, Bull, & Parker, 2015; Hate et al., 2015; Jao et al., 2015; Merson et al., 2015). We have argued that together the analyses from each of these five settings suggest four key *prima facie* principles or requirements for the assessment of the extent to which a proposed model of data sharing exemplifies good practice and is likely to be capable of commanding the confidence of key stakeholders in such settings. The four requirements are as follows: the likelihood that sharing will contribute to scientific knowledge relevant to health care, minimal risk of harm, fairness and reciprocity, and trust. We have also explored what might be important considerations arising out of this research in relation to four areas of practice: consent, governance, data-sharing policy, and capacity building.

We began this article by making reference to debate about the importance, in successful and appropriate scientific research, of a reasonable degree of congruence between what the public and research participants consider themselves to have given scientists as a “social license,” and the mandate scientists and other research actors understand themselves to have been given. Our research suggests that the social license for data sharing in low-income settings might best be understood in terms of the criteria outlined above. Clearly, the findings of our research are not necessarily generalizable to other settings or to other forms of research in the settings studied. They do nonetheless suggest important considerations for further discussion and potential avenues for future research on this topic elsewhere. We conclude this article with some final reflections on the implications of our findings for education and research.

## Educational Implications

The majority of stakeholders in this study had very limited experience of sharing individual-level health research data: Even senior stakeholders had little experience of sharing data with secondary researchers who were not already known to them. If data sharing is to be promoted in such settings, resources outlining potential benefits of data sharing, ethical issues, and concerns that may arise when sharing data and means of appropriately responding to these are needed. Such resources are needed to inform researchers who are developing data-sharing plans for grant applications and protocols submitted for ethical review, ethical committees reviewing data-sharing proposals, data-access managers, and community advisory boards seeking to represent participants’ and community interests.

To assist in addressing these educational needs, we have developed an open access online resource focusing on ethics and best practices in sharing individual-level data in low- and middle-income settings, which is available at https://bioethicsresearchreview.tghn.org/research-data-sharing/. A core component is a free online-certified course designed to support researchers and research ethics committees seeking to build capacity in data-sharing ethics. Additional resources include a compilation of relevant guidance, policies, and peer-reviewed publications, as well as links to data-sharing repositories from a range of settings. Open-access discussion groups and blogs hosted within the site enable stakeholders from around the world to further develop the resource and to converse about ethical aspects of data sharing and best practices in addressing these.

## Research Agenda

As discussed above, the systematic literature review undertaken as part of this study was unable to identify any prior empirical research into stakeholders’ perspectives about ethical aspects of sharing individual-level data from clinical and public health in low- and middle-income settings (Bull, Roberts & Parker, 2015). Consequently, we believe that more research is needed on all aspects of data sharing in low- and middle-income settings. The research presented here and in the other papers collected in this special issue is an important contribution to the debate and suggests a number of avenues for additional research. It is clear that further research into stakeholders’ perspectives and data-sharing practices is required in these and other settings in low- and middle-income countries where there is significant research activity. In addition to further research on the views of key stakeholders about the key requirements for good data-sharing practice, there is also a need for research on the development, implementation, and evaluation of different models of data sharing. Research will also need to be conducted on the development and evaluation of the key areas discussed in the previous sections: consent, governance, policy, and capacity building. Research which maps and quantifies the extent and forms of emerging data-sharing practices and assesses their impact on levels of scientific activity will be important. Further research will also be required to map and track public and expert opinion and experience over time.

The research areas outlined above are important for several reasons. These include the need to establish the conditions and requirements for effective and appropriate data-sharing practices capable of commanding the trust and confidence of relevant parties. It is also important because many of the arguments in favor of data sharing as a promoter of scientific progress and the production of knowledge are empirical claims. Our research suggests that for data sharing to be effective and sustainable, several other social and ethical requirements need to be met. This, in turn, suggests that an effective model of data sharing will be one in which considered judgments will need to be made about how best to achieve scientific progress, minimize risks of harm, promote fairness and reciprocity, and build and sustain trust.
